# Age at natural menopause in three Central and Eastern European urban populations: The HAPIEE study

**DOI:** 10.1016/j.maturitas.2013.02.008

**Published:** 2013-05

**Authors:** U. Stepaniak, K. Szafraniec, R. Kubinova, S. Malyutina, A. Peasey, H. Pikhart, A. Pająk, M. Bobak

**Affiliations:** a Department of Epidemiology and Population Studies, Jagiellonian University Medical College, Krakow, Poland; b National Institute of Public Health, Prague, Czech Republic; c Institute of Internal Medicine, Russian Academy of Medical Sciences, Novosibirsk, Russia; d Department of Epidemiology and Public Health, University College London, London, United Kingdom

**Keywords:** Age at menopause, Natural menopause, Lifestyle, Socioeconomic, Smoking, Diet, Central and Eastern Europe

## Abstract

**Objectives:**

To investigate the age at menopause in three urban populations in Central and Eastern Europe and to assess whether the (suspected) differences can be explained by a range of socioeconomic, reproductive and behavioural factors.

**Methods:**

The Health, Alcohol and Psychosocial factors in Eastern Europe (HAPIEE) Study examined random samples of populations aged 45–69 years in Novosibirsk (Russia), Krakow (Poland) and six Czech towns. Participants completed a questionnaire and attended an examination in clinic. A total of 12,676 of women were included in these analyses.

**Results:**

The median age at menopause was 50 years in Novosibirsk, 51 years in Czech towns and 52 years in Krakow; the Cox regression hazard ratios of menopause, compared with Krakow, were 1.47 (95% CI 1.40–1.55) for Novosibirsk and 1.10 (1.04–1.16) for Czech women. In multivariate analyses, higher education, using vitamin and mineral supplements and ever use of oral contraceptives were associated with later menopause, while smoking, abstaining from alcohol and low physical activity were associated with earlier menopause. These factors, however, did not explain the differences between populations; the multivariate hazard ratios of menopause, compared with Krakow, were 1.48 (1.40–1.57) for Novosibirsk and 1.11 (1.05–1.17) for Czech women.

**Conclusions:**

In this large population based study, differences in age at menopause between Central and Eastern Europe populations were substantial and unexplained by a range of risk factors. Associations of age at menopause with risk factors were largely consistent with studies in other populations.

## 1 Introduction

The age of the onset of menopause varies between populations and, even more so, among women within populations. However, it is unclear whether the differences could be explained by socioeconomic and lifestyle characteristics [1], [2], [3], [4]. The societal transition in Central and Eastern Europe caused dramatic changes in socioeconomic status, health behaviours and lifestyle; these changes are likely to have impact on health status and in women on the age at menopause. Age at menopause is an important health indicator; total mortality is reduced by some 2% with the increase of each year of age at menopause, and it has been suggested that it may serve as a marker of population health [5], [6], [7]. In the 1990s, age at menopause in Russia was lower than in other countries of Central and Eastern Europe [3]. Studies in western populations suggest that the age at menopause increased in recent decades [2], [7], [8], and this shift may be related to changes of socioeconomic conditions and lifestyle, particularly among young women.

In addition to genetic factors [1], [7], variables related to menstrual and reproductive history, such as parity or menstrual cycle length are known to influence age at menopause [1], [2], [3], [9], [10], [11], [12], although studies on oral contraceptives (OC) have produced mixed results [9], [11], [12], [13]. A number of lifestyle and socioeconomic factors have also been studied in relation to the onset of menopause. There is a consistent observation that cigarette smoking is related with earlier age at menopause [1], [2], [8], [9], [10], [11], [12], [13], [14], [15], [16], [17], [18], probably reflecting the cytotoxic effect on ovaries and its anti-estrogenic effects [1]. But the evidence is less clear on the role of other factors, such as education [1], [9], [11], [12], [15], [16], [18], marital status [9], [12], [18], [19], [20], physical activity [1], [2], [8], [9], [13], [14], [17], or dietary habits (e.g. coffee consumption [18], [19], [21] or intake of fruits and vegetables [14], [17], [22]). Poor socioeconomic status was associated with earlier menopause in previous studies [1], [12], [15], possibly reflecting exposure to psychosocial and physical stress or of unhealthy behaviours. Alcohol consumption may be estrogenic [21] but the evidence on its association with age at menopause is ambiguous [9], [13], [14], [16], [18], [21], [23], [24]. Oestrogen conversion in fatty tissue leads to higher circulating levels of estrogens [1], [10] but the relationship between obesity and age at menopause in previous studies has been inconsistent [1], [9], [10], [13], [14], [17].

Since both the onset of menopause and the distribution of known and suspected risk factors differ between populations, it is useful to investigate the risk factors for onset of menopause across populations. One particular region where studies of menopause onset have been sparse is Central and Eastern Europe [2], [9], [25]. In this paper, we examined the relationship between age at natural menopause and socioeconomic and lifestyle characteristics in women from Czech Republic, Russia and Poland. The secondary aim was to investigate the extent to which differences in age of onset of natural menopause between populations can be explained by lifestyle factors and socioeconomic status.

## 2 Methods

### 2.1 Study populations and study subjects

These analyses used data from the baseline survey of the cohort HAPIEE Study (Health, Alcohol and Psychosocial factors In Eastern Europe), conducted in Novosibirsk (Russia), Krakow (Poland) and six Czech towns (Havirov/Karvina, Jihlava, Usti nad Labem, Liberec, Hradec Kralove and Kromeriz). Details of the study design have been published elsewhere [26]. Briefly, random samples of men and women aged 45–69 years were selected from urban population registers (electoral lists in Russia). In Krakow and Czech towns, subject were first visited at home to complete questionnaire and then invited to a clinic for examination; in Novosibirsk, both the questionnaires and examinations have been completed in a clinic. The response rates were 61% in Novosibirsk and Krakow and 55% in Czech towns (overall response rate 59%) [26]. The study was approved by the University College London Hospital ethics committee and by the local ethics committee in every participating centre. All participants gave written informed consent.

### 2.2 Measurements

Data on menopausal status, use of hormonal contraceptives and hormone replacement therapy (HRT), lifestyle factors (smoking, physical activity, supplementation with vitamins and minerals, frequency of alcohol consumption), demographic and socioeconomic characteristics (age, gender, education, marital status) were collected using a standard questionnaire. Reliability of local version of the questionnaire was assessed by back-translation into English [26].

Natural menopause was defined according to WHO criteria, as the permanent cessation of menstruation recognized as at least 12 consecutive months of amenorrhea [27]. In our study, this was based on questions on the presence of regular/irregular periods, age of last period, and whether the menopause was natural or surgical.

Education (classified into 5 categories) was dichotomized into university vs. secondary or lower. Marital status was classified into 4 categories: single, married/cohabiting, divorced/separated, and widowed. Smoking was classified into 4 categories: non-smokers (less than 1 cigarette per day); 1–9 cigarettes per day; 10–19 cigarettes per day; and 20+ cigarettes per day. Frequency of alcohol consumption during past 1 year was classified into 4 categories: never; 1–11 times per year (low); 3 times per month to 1–4 times per week (moderate); and 5+ times per week (everyday). Body mass index (BMI, kg/m^2^) was categorized into 5 groups: underweight (BMI <20); normal (BMI 20.0–24.9); overweight (BMI 25.0–29.9); obesity (BMI 30.0–34.9); and severe obesity (BMI ≥35.0). Leisure time physical activity (LTPA), assessed by duration of the time spent on leisure physical activity during the average week; was classified into 3 categories: no LTPA (0 h per week); moderate LTPA (1–5 h per week); and high LTPA (6+ h per week). The use of supplement vitamins or minerals, hormonal contraceptives (currently or in the past) and ever using HRT were dichotomized (yes/no).

Food frequency questionnaire [28] was used to assess consumption of coffee, fruits and vegetables. Frequency of coffee consumption was classified as no consumption; one cup per day and 2 or more cups per day. Fruits and vegetables consumption was classified into 3 categories: low (<1 portion per day), moderate (1–2 portions per day); and high (3+ portions per day).

### 2.3 Statistical analysis

Of 15,323 women who participated in the study, 2342 women with surgical and other non-natural menopause and 305 women who did not answer questions on menstruation history were excluded, leaving 12,676 women with full data for analysis. The median age at natural menopause in Czech, Russia and Poland was estimated by Kaplan–Meier survival method and log-rank test was used to test differences between populations. In the survival analysis women were considered to have entered the study at birth; if they reached natural menopause during the followed-up, they were considered completed cases, otherwise (still menstruating at examination), they were censored. The endpoint time was defined as age of menopause for completed and age at interview for censored observations.

The relationships between age at menopause and lifestyle variables were assessed by Cox proportional hazards regression modelling. Proportionality assumptions were investigated separately in each country by including into regression models interaction of analyzed risk factor and logaritmically transformed time to the event. In the models, there were significant interactions between ln(time) and age in Novosibirsk and Czech towns, ln(time) and education in Czech towns and Krakow, ln(time) and smoking in all countries, indicating that the proportional hazards assumptions may be invalid for those factors. However, inspection of the martingale and deviance residuals for all significant covariates revealed no outliers. It was therefore concluded that the non-proportionality made no difference to the interpretation of the data for all sample size [29].

The analysis was conducted in two steps. First, each single socioeconomic and lifestyle factor was included separately as independent variable. Second, variables which were significantly associated with age at menopause in univariate analyses were included into the multivariate model. The interactions between baseline characteristics and study population were assessed in the Cox models by the likelihood ratio tests (comparing models with and without the interaction term). As no interactions were detected (all *p* > 0.05), pooled results are presented. SAS software (version 9.1; SAS Institute, Cary, NC) and Statistica version 10.0 software were used; *p* < 0.05 was set as the level of statistical significance. A hazard ratio less than 1.0 indicates that natural menopause for the exposed group occurred later than in the referent group and hazard ratio more than 1.0 means that natural menopause for the exposed group occurred earlier than in the referent group.

## 3 Results

**Fig. 1:**
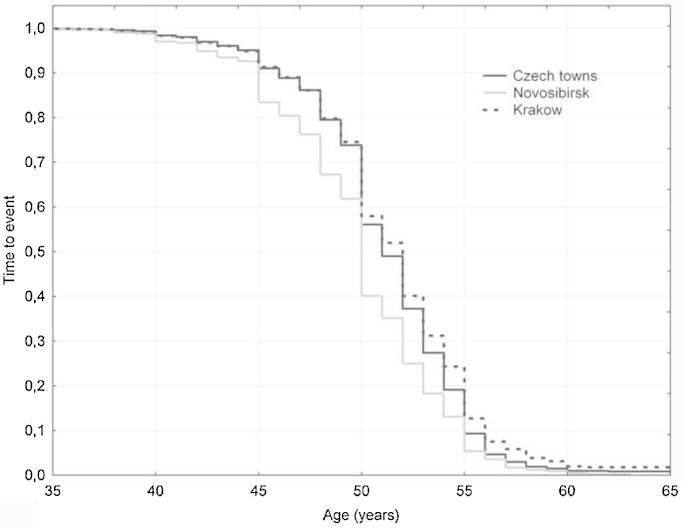
Survival curves for age at natural menopause in studied populations; Kaplan–Meier estimates.

Among 12,676 women with valid data, 9094 (72%) were classified as having reached natural menopause while 3582 (28%) were pre-menopausal. Descriptive statistics by population are presented in Table 1. The proportion of women with university education was similar in Novosibirsk and Krakow and lowest in Czech towns. Russian women had the lowest frequency of being married, use of HRT and smoking and the highest prevalence of obesity and physical inactivity. Median age at menopause was the lowest in Russia (50 years), in Czech Republic it was 51 years and the highest was in Poland (52 years) (*p* < 0.0001); the survival plot by population is shown in Fig. 1.

**Table 1:** Descriptive characteristics of the study populations and median (inter-quartile range, IQR) of age of onset of menopause by covariates.

Variables	Czech towns		Novosibirsk		Krakow		All	
No. of women (% having natural menopause)	3689 (71.4%)		4394 (77.2%)		4593 (66.9%)		12,676 (71.7%)	
Age at natural menopause, median (IQR)	51.0 (49.0; 54.0)		50.0 (48.0; 52.0)		52.0 (49.0; 54.0)		51.0 (49.0; 54.0)	
Socioeconomic and lifestyle characteristics, %, age at natural menopause, median (IQR)	%	Median (IQR)	%	Median (IQR)	%	Median (IQR)	%	Median (IQR)
Education								
Secondary or lower	89.7	51 (49; 54)	73.6	50 (47; 52)	72.7	51 (49; 54)	77.9	50 (48; 53)
University	10.3	52 (50; 55)	26.4	50 (48; 53)	27.3	52 (50; 55)	22	52 (50; 54)
Marital status								
Single	2.6	50 (49; 53)	4.8	50 (48; 53)	7.2	51 (50; 54)	5	51 (48; 54)
Married or cohabited	68.3	52 (50; 54)	59.1	50 (48; 53)	66.1	52 (50; 55)	64.3	51 (49; 54)
Divorced	15	51 (48; 54)	14.4	50 (48; 52)	9.1	52 (49; 54)	12.7	50 (48; 53)
Widowed	14.1	51 (49; 53)	21.7	50 (47; 52)	17.6	51 (49; 54)	18	50 (48; 53)
Smoking (cigarettes per day)								
0	78.1	52 (50; 54)	90.3	50 (48; 52)	73.7	52 (50; 55)	80.7	51 (49; 54)
1–9	5.4	51 (49; 54)	5.1	50 (47; 53)	4.7	52 (50; 55)	5.1	51 (48; 54)
10–19	12	50 (48; 53)	3.2	50 (47; 52)	11.1	51 (49; 53)	8.6	50 (48; 53)
20 or more	4.5	50 (48; 53)	1.4	50 (46; 53)	10.5	50 (48; 54)	5.6	50 (48; 53)
BMI (kg/m^2^)								
<20	2.1	50 (48; 53)	2	50 (46; 52)	2.3	51 (48; 53)	2.1	50 (48; 53)
20–24.9	28	52 (50; 54)	16.7	50 (48; 52)	26.4	52 (50; 55)	23.1	51 (49; 54)
25–29.9	38.8	51 (49; 54)	34.8	50 (48; 53)	38	52 (50; 54)	37	51 (49; 53)
30–34.9	21.2	51 (49; 54)	28.6	50 (47; 52)	23.5	51 (49; 54)	24.9	50 (48; 53)
35 or greater	9.9	51 (48; 54)	17.8	50 (48; 53)	9.8	52 (49; 55)	12.9	50 (48; 54)
Physical activity (h/week)								
0	30.6	51 (49; 54)	73.2	50 (48; 52)	30.9	51 (49; 54)	45.9	50 (48; 53)
1–5	41.6	52 (49; 54)	11	50 (48; 53)	32.2	52 (50; 55)	27.4	52 (49; 54)
6 or more	27.7	51 (49; 53)	15.8	50 (48; 52)	36.9	52 (50; 54)	26.7	51 (49; 54)
Frequency of alcohol consumption								
Never	18.1	50 (48; 53)	17.7	50 (47; 52)	38	51 (48; 54)	24.4	50 (48; 53)
Low	32.2	51 (49; 54)	55.7	50 (48; 52)	31.7	52 (50; 55)	40.9	50 (48; 53)
Moderate	45.2	52 (50; 54)	26.3	50 (48; 53)	29.1	52 (50; 55)	32.9	51 (49; 54)
Everyday	4.5	51 (49; 54)	0.3	50 (46; 51)	1.3	51 (48; 52)	1.9	51 (49; 54)
Supplementation with vitamins and minerals								
No	39.6	51 (49; 54)	59.4	50 (47; 52)	45.3	51 (49; 54)	48.6	50 (48; 53)
Yes	60.4	52 (49; 54)	40.6	50 (48; 53)	54.7	52 (50; 55)	51.4	51 (49; 54)
Hormonal contraceptives								
No	71.3	51 (49; 54)	91.3	50 (48; 52)	85.8	52 (49; 54)	83.5	50 (48; 53)
Yes	28.7	52 (50; 54)	8.7	51 (48; 53)	14.2	52 (50; 55)	16.5	52 (50; 55)
HRT								
No	81.2	51 (49; 54)	93.2	50 (48; 52)	74	52 (49; 54)	82.9	50 (48; 53)
Yes	18.8	52 (50; 55)	6.8	50 (48; 53)	26	52 (49; 55)	17.1	52 (49; 55)
Coffee (cups/day)								
0	16.3	51 (49; 53)	60.9	50 (48; 52)	27.2	52 (49; 54)	35.7	50 (48; 53)
1	36.2	51 (49; 54)	26.3	50 (48; 53)	45.5	52 (49; 54)	36.1	51 (49; 54)
2–3 or more	47.6	51 (49; 54)	12.8	50 (47; 53)	27.3	52 (50; 54)	28.1	51 (49; 54)
Fruits (portions/day)								
0	28.8	51 (49; 54)	71.3	50 (48; 52)	22.9	51 (49; 54)	42.2	50 (48; 53)
1–2	38.4	51 (49; 54)	22.3	50 (48; 53)	55	52 (49; 54)	38.4	51 (49; 54)
3 or more	32.8	51 (49; 54)	6.4	50 (48; 53)	22.1	52 (49; 54)	19.4	51 (49; 54)
Vegetables (portions/day)								
0	61.3	51 (49; 54)	14	50 (48; 53)	68	52 (49; 54)	46.4	51 (49; 54)
1–2	21.2	51 (49; 54)	43.8	50 (48; 53)	20.2	52 (49; 55)	29.1	50 (48; 53)
3 or more	17.5	52 (50; 54)	42.2	50 (47; 52)	11.8	52 (49; 54)	24.5	50 (48; 53)

The univariate relationships between age at menopause and socioeconomic and lifestyle characteristics are presented in Table 2. University education was associated with lower risk of menopause while widowed women experienced earlier menopause compared with married or cohabited. Smoking and low LTPA were associated with earlier age at menopause. In all populations, women who reported to be never drinkers experienced earlier menopause compared with alcohol consumers. Age at menopause was not significantly associated with BMI, except of Russian women with BMI of 35 or greater who experienced menopause later then women with normal weight; however, due to small number of such women in Krakow and Czech samples, the interaction with study centre was not statistically significant. Using vitamin or mineral supplements, hormonal contraception and HRT were associated with later menopause. Frequency of consumption of neither coffee nor fruits/vegetables was significantly associated with age at menopause.

**Table 2:** Univariate hazard ratio (95% CI) of age at menopause according to socioeconomic and lifestyle factors by study population.

Socioeconomic and lifestyle characteristics	Czech towns	Novosibirsk	Krakow	All
	HR (95% CI)	HR (95% CI)	HR (95% CI)	HR (95% CI)^a^
Education				
Secondary or lower	1.00	1.00	1.00	1.00
University	0.77 (0.67–0.88)	0.86 (0.79–0.93)	0.77 (0.71–0.83)	0.81 (0.77–0.85)
Marital status				
Single	1.19 (0.93–1.53)	0.99 (0.84–1.17)	1.11 (0.96–1.28)	1.07 (0.97–1.18)
Married or cohabited	1.00	1.00	1.00	1.00
Divorced	1.04 (0.92–1.16)	1.08 (0.97–1.19)	1.07 (0.94–1.22)	1.06 (1.00–1.14)
Widowed	1.14 (1.03–1.26)	1.21 (1.12–1.31)	1.18 (1.08–1.29)	1.19 (1.13–1.25)
Smoking (cigarettes per day)				
0	1.00	1.00	1.00	1.00
1–9	0.97 (0.80–1.17)	1.07 (0.90–1.27)	0.98 (0.81–1.17)	1.01 (0.91–1.12)
10–19	1.29 (1.14–1.46)	1.14 (0.93–1.40)	1.26 (1.12–1.42)	1.24 (1.14–1.34)
20 or more	1.17 (0.96–1.42)	1.03 (0.76–1.41)	1.44 (1.28–1.63)	1.30 (1.18–1.43)
BMI (kg/m^2^)				
<20	1.04 (0.73–1.47)	1.08 (0.82–1.41)	1.05 (0.77–1.42)	1.07 (0.90–1.27)
20–24.9	1.00	1.00	1.00	1.00
25–29.9	1.07 (0.96–1.20)	0.96 (0.86–1.06)	0.99 (0.90–1.10)	1.01 (0.95–1.07)
30–34.9	1.11 (0.98–1.26)	0.98 (0.88–1.09)	1.08 (0.97–1.20)	1.05 (0.99–1.12)
35 or greater	1.15 (0.99–1.34)	0.86 (0.76–0.97)	1.03 (0.89–1.18)	0.97 (0.90–1.05)
Physical activity (h/week)				
0	1.12 (1.02–1.23)	1.14 (1.01–1.28)	1.14 (1.04–1.25)	1.13 (1.07–1.20)
1–5	1.00	1.00	1.00	1.00
6 or more	1.15 (1.05–1.27)	1.09 (0.95–1.25)	0.99 (0.90–1.08)	1.06 (1.00–1.13)
Frequency of alcohol consumption				
Never	1.20 (1.08–1.33)	1.11 (1.01–1.21)	1.22 (1.11–1.33)	1.16 (1.10–1.23)
Low	1.00	1.00	1.00	1.00
Moderate	0.96 (0.88–1.06)	0.92 (0.85–1.00)	1.01 (0.90–1.12)	0.95 (0.90–1.01)
Everyday	1.01 (0.82–1.24)	0.73 (0.30–1.76)	1.24 (0.84–1.83)	1.02 (0.86–1.21)
Supplementation with vitamins and minerals				
No	1.00	1.00	1.00	1.00
Yes	0.92 (0.85–1.00)	0.88 (0.82–0.94)	0.91 (0.84–0.97)	0.90 (0.86–0.94)
Hormonal contraceptives				
No	1.00	1.00	1.00	1.00
Yes	0.84 (0.76–0.92)	0.79 (0.68–0.91)	0.71 (0.63–0.80)	0.79 (0.74–0.84)
HRT				
No	1.00	1.00	1.00	1.00
Yes	0.81 (0.73–0.90)	0.86 (0.75–1.00)	0.85 (0.79–0.93)	0.85 (0.80–0.90)
Coffee intake (cups/day)				
0	1.00	1.00	1.00	1.00
1	0.96 (0.86–1.08)	0.96 (0.89–1.04)	1.01 (0.93–1.10)	0.98 (0.93–1.03)
2–3 or more	0.98 (0.88–1.09)	0.96 (0.86–1.07)	0.97 (0.87–1.07)	0.97 (0.91–1.02)
Fruits (portions/day)				
0	1.02 (0.92–1.13)	1.05 (0.97–1.14)	1.04 (0.95–1.14)	1.04 (0.99–1.09)
1–2	1.00	1.00	1.00	1.00
3 or more	1.02 (0.93–1.12)	0.92 (0.79–1.07)	0.97 (0.88–1.06)	0.99 (0.93–1.05)
Vegetables (portions/day)				
0	0.99 (0.89–1.10)	1.01 (0.91–1.12)	1.07 (0.97–1.17)	1.03 (0.97–1.09)
1–2	1.00	1.00	1.00	1.00
3 or more	0.99 (0.87–1.12)	1.07 (1.00–1.15)	1.05 (0.92–1.21)	1.05 (0.99–1.11)

a Adjusted for population.

Results of multivariate models in pooled data are shown in Table 3. The differences between populations were substantial: Novosibirsk women had approximately 50% higher risk of menopause than women in Krakow, and this excess was similar in crude and multivariate models. Higher level of education was related with a 15% lower risk of menopause; smoking 20+ cigarettes per day increased risk of menopause by 39%; never drinkers had 10% higher risk of menopause, BMI of 35 or greater was associated with a 12% lower risk of menopause (largely due to the Novosibirsk sample); and hormonal contraception decreased the risk of menopause by 13%. Marital status, low LTPA, vitamin and mineral supplements, HRT were not strongly related to menopause, although some of the associations were of borderline significance. Results were virtually identical when data were restricted to women who never used HRT (not shown in table; results available on request).

**Table 3:** Adjusted hazard ratio (95% CI) of age at natural menopause by socioeconomic and lifestyle factors in the pooled data.

Socioeconomic and lifestyle characteristics	Model 1	Model 2
	HR (95% CI)^a^	HR (95% CI)^b^
Population		
Krakow	1.00	1.00
Czech towns	1.10 (1.04–1.16)	1.11 (1.05–1.17)
Novosibirsk	1.47 (1.40–1.55)	1.48 (1.40–1.57)
Education		
Secondary or lower	1.00	1.00
University	0.83 (0.79–0.88)	0.85 (0.81–0.90)
Marital status		
Single	1.09 (0.98–1.20)	1.10 (0.99–1.21)
Married or cohabited	1.00	1.00
Divorced	1.08 (1.01–1.16)	1.06 (0.99–1.13)
Widowed	1.08 (1.02–1.14)	1.04 (0.99–1.10)
Smoking (per day)		
No	1.00	1.00
1–9	1.11 (1.00–1.23)	1.10 (0.99–1.23)
10–19	1.34 (1.24–1.45)	1.31 (1.21–1.42)
20 or more	1.43 (1.29–1.57)	1.39 (1.26–1.54)
BMI (kg/m^2^)		
<20	1.09 (0.92–1.30)	1.02 (0.85–1.21)
20–24.9	1.00	1.00
25–29.9	0.97 (0.92–1.03)	0.97 (0.92–1.03)
30–34.9	1.00 (0.93–1.06)	0.99 (0.93–1.06)
35 or greater	0.91 (0.84–0.98)	0.88 (0.82–0.96)
Physical activity (h/week)		
0	1.10 (1.04–1.16)	1.06 (1.00–1.12)
1–5	1.00	1.00
6 or more	1.04 (0.98–1.10)	1.03 (0.97–1.09)
Frequency of alcohol consumption		
Never	1.11 (1.05–1.17)	1.10 (1.04–1.16)
Low	1.00	1.00
Moderate	0.99 (0.94–1.05)	1.00 (0.95–1.06)
Everyday	1.05 (0.88–1.25)	1.05 (0.88–1.25)
Supplementation with vitamins and minerals		
No	1.00	1.00
Yes	0.93 (0.89–0.97)	0.96 (0.92–1.00)
Hormonal contraceptives		
No	1.00	1.00
Yes	0.85 (0.80–0.91)	0.87 (0.81–0.93)
HRT		
No	1.00	1.00
Yes	0.90 (0.85–0.95)	0.95 (0.89–1.01)

a Adjusted for: age, population.

b Adjusted for: age, population, education, marital status, smoking, BMI, physical activity, alcohol consumption, supplementation with vitamins and minerals, hormonal contraceptives, HRT.

## 4 Discussion

Data from this large study in three urban populations of Central and Eastern Europe indicated that age at natural menopause, which is natural stage of ageing process, significantly differs between studied populations and this difference was not explained by other risk factors. The associations between other risk factors and menopause are consistent with observations in other populations.

### 4.1 Study limitations and strengths

The main limitation of our study is the cross-sectional nature of the data, which prevents direct investigation of causal relationship between lifestyle factors and age at menopause. It is possible that during menopausal transition women have changed some of their lifestyle habits, such as physical activity or diet. Similarly, weight gain, often observed after menopause, can obscure the association with obesity.

The second main limitation is the relatively low response rate (59%), although this is similar to many current population-based studies. Analysis of the Krakow sample showed that participants of the baseline survey had lower mortality than non-respondents [30]. Although data for such analysis were not available for Novosibirsk and Czech towns, previous studies have shown that, compared to respondents in population based studies, non-respondents are less healthy, have lower socioeconomic position and higher prevalence of unhealthy life styles. It is therefore likely that the participants were healthier than the general populations from which they were selected; in this case the mean age of onset of menopause in the general population may be lower. However, it is less likely that response rate would also bias the associations between age at menopause and studied factors.

Third, associations examined in our study could be potentially confounded by other factors, such as reproductive history (e.g. parity). Unfortunately, these data have not been collected. Participants only reported ever use of HRT, and some of the women may have been users during the study. However, excluding ever users from the analyses did not materially change the results.

Finally, as in most other studies, data on the last menstrual period and nature of menopause were self-reported, and could be affected by imprecise recall. A similar problem may affect recall of lifestyle behaviours. However, it is most likely that the misclassification would be non-differential, which would lead to underestimating the strength of the associations.

On the other hand, large random population samples of women in Central and Eastern Europe have not been studied in this detail before. The centrally designed protocol ensures good quality of data and comparability across populations. Despite the issue with self-report, is it unlikely that our results are confounded by surgical or other non-natural induced menopause.

Another strength is the use of the Cox regression; this approached is more efficient than logistic regression because, since it models the time to event, it is more statistical powerful than using dichotomous outcome in logical regression. Nevertheless, we also conducted analyses using logistic regression and found that the results, and effect sizes, were similar.

### 4.2 Differences between populations

We found that the age at menopause was lower in Novosibirsk than in the other two populations. The low age at menopause in Russia is consistent with other available data [3]. Simultaneous adjustment for all available covariates did not reduce the differences between populations. However, this lack of explanatory power may reflect the fact that Novosibirsk women had low prevalence of both harmful factors (smoking) and protective factors (oral contraceptives and HRT). It is likely that in multivariate models, the effects negative/positive confounders (mediators) cancelled out and the total population effect did not change. Although our study did not identify factors that may cause the difference between populations, the lower age at menopause in Russian women is consistent with their lower life expectancy, compared with Polish and Czech women. This ecological pattern confirms the notion that age at menopause may serve as an indicator of general health status [1], [6].

### 4.3 Associations with covariates

Associations of individual-level covariates with menopause are largely consistent with previous studies. Consistently with earlier findings, higher education, an indicator of socioeconomic status, was independently related to later age at menopause [8], [9], [14], [16], [19]. Although we found no overall effect of marital status, widowed women in Novosibirsk experienced earlier menopause than married women (adjusted HR 1.10, not shown in tables); this could reflect lower family social status or unhealthy lifestyle which may have contributed of early deaths of many Russian men resulting in very high proportion of widowed women in the Russian population [31]. Earlier age at menopause of widowed women has previously been observed in Norway and in US [12], [18].

The association of current smoking with earlier age at natural menopause is consistent with the literature studies [2], [9], [10], [11], [15], [16], [17]. Given the persistently high prevalence of female smokers in many countries (e.g. Poland [32]), and the increasing smoking rates in young women in Russia [33], tobacco is a crucial lifestyle factor in Central and Eastern Europe.

In Russia, where the prevalence of obesity is very high [34], severe obesity was associated with later age at menopause, while underweight, overweight and obesity were not. BMI was related with age at menopause in the Multiethnic Cohort Study [10] and in Shanghai Women's Health Study [14] but not in other investigations [13], [17].

In our data, women who did not drink alcohol experienced earlier menopause than drinkers, while among drinkers the frequency of consumption was not related with menopause. Pro-estrogenic effect of moderate alcohol intake without dose–response effect have previously been reported [16], [21], [23]. However, abstaining from alcohol may be related with other traits (poor health, alcohol dependence) which may confound this observation; longitudinal data would be required to address this important question.

The WHO MONICA Project has shown that Eastern Europe has very low rates of OC and HRT use [35], and in this study, the use of hormonal contraceptives was rare, especially in Russia. Consistently with previous studies, hormonal contraception was associated with later age at menopause [9], [12]. However, this association was not confirmed in prospective studies [11], [13] and, again, longitudinal data would be needed. Unfortunately, we did not have information on type and time of contraception use. HRT, although not associated with age at menopause in our study, can mask the natural cessation of menses [1] and potentially may be a source of classification bias. However, exclusion of ever users did not change the effect estimates.

The three nutrition markers included in this study (coffee, fruits and vegetables, and vitamin and mineral supplements) were not strongly associated with menopause in multivariate models. The literature on these factors is not entirely consistent. Coffee consumption was inversely associated with menopause in Japanese women [19] but not in other studies [18], [21]. In the EPIC study, higher intake of vegetables was related with earlier menopause [17] but in other prospective studies fruits and vegetables were associated with later menopause [14], [22]. The use of nutritional supplements, which is likely to reflect other personal characteristics [36], have not been specifically studied in relation to menopause but our results do not suggest a strong association.

Socioeconomic and lifestyle factors may play an important role in changing the onset of menopause, and important process which is associated with future health outcomes [7]. Modification of risk factors, such as smoking, may postpone the onset of menopause more closely to the biologically determined individual time of menopause. However, smoking and other risk factors did not explain the variation in age at menopause between our three Central and Eastern European study populations, and it remains to be seen whether individual-level risk factors for menopause are also the main determinants of menopause at the population level.

## Contributors

US participated in writing of the manuscript, data analysis and drafted the paper. KS was involved in statistical analyses. AP and MB critically reviewed and modified the first draft. All authors commented to subsequent drafts of the paper. MB, AP, HP, SM, RK and AP were responsible for the design and execution of the Project. AP, SM and RK were local principal investigators responsible for the data collection. Final version of the manuscript was approved by all authors.

## Competing interests

The authors declare no conflicts of interest.

## Funding

The study has been funded by the Wellcome Trust (grants 064947/Z/01/Z and 081081/Z/06/Z), US National Institute on Ageing (grant 1R01 AG23522-01) and the MacArthur Foundation Initiative on Social Upheaval and Health (award 71208).

## Ethical approval

The study was approved by the University College London Hospital ethics committee and by the local ethics committee in every participating centre. All participants gave written informed consent.
